# Brodie's Abscess of the Tibia Presenting as Bilateral Hip Pain: A Diagnostic Challenge

**DOI:** 10.1002/ccr3.71484

**Published:** 2025-11-16

**Authors:** Suleiman Sbeih, Yazan Ahmad, Mohammed Boji, Asma Mahajnah, Fawzi Abunejma, Mohammad Alashqar

**Affiliations:** ^1^ Faculty of Medicine Al‐Quds University Abu Dis Palestine; ^2^ Al‐Ahli Hospital Hebron Palestine; ^3^ Faculty of Medicine and Health Sciences An‐Najah National University Nablus Palestine

**Keywords:** Brodie's abscess, infection, subacute osteomyelitis, tibial lesion

## Abstract

Referred hip pain in a child could mask Brodie's abscess of the tibia. Recognition of this unusual presentation and prompt MRI assessment are very important to avoid misdiagnosis and to appropriately guide surgical and antibiotic management.

## 1 Introduction

Brodie's abscess is a subacute form of osteomyelitis manifesting as a pus collection within the bone, frequently with insidious onset [1, 2]. It was identified in 1832 by Sir Benjamin Brodie [3]. Brodie's abscesses occur in males more than females and typically occur in patients under the age of 25 [1], when subacute or chronic osteomyelitis affects the metaphysis of long bones (tibia, fibula and radius). It typically results from hematogenous spread, though trauma can also precipitate infection [4]. The most common pathogen is *Staphylococcus aureus*; other gram‐positive and gram‐negative bacteria, including *Pseudomonas aeruginosa* , Klebsiella and other gram‐negative rods may be seen. However, cultures may be sterile in up to half of cases [5].

Brodie's abscess patient typically presented with insidious onset of mild to moderate pain lasting for several weeks to months, with or without fever [6]. Diagnosis of Brodie abscess is challenging as patients may present with minor symptoms and unrevealing laboratory studies [7]. Biopsy for culture and histologic examination is often needed in addition to radiography to confirm the diagnosis [8]. The preferred method of treatment is surgical debridement followed by a prolonged course of parenteral antibiotics, and the results are typically favorable [9]. The common presentation of Brodie's abscess is characterized by the presence of localized pain over the involved bone, but there have been reports of atypical presentations with referred pain, although these are still rare occurrences, especially among young children [1, 6]. For instance, van der Naald et al. [6], Vishwakarma et al. [10], and Chen et al. [9] have presented cases where the localization of the pain was misleading and not the actual site of infection, which resulted in delays in diagnosis. Our case adds an unusual case of Brodie's abscess of the tibia being primarily expressed as bilateral hip pain, thus highlighting the diagnostic difficulty of referred pain in children.

## 2 Case History/Examination

A 3‐year‐old male patient presented to the pediatric ward with a 2‐weeks history of bilateral hip pain mainly at night. One week later he developed a left‐sided limp that fluctuated in severity from barely noticeable to hardly being able to bear weight. Three days prior to admission, the patient started complaining of fever and vomiting after each feeding, associated with poor oral intake. Twelve days prior to his admission the parents sought medical advice from an outpatient doctor who in turn gave him Augmentin 75 mg/kg and Ibuprofen 10 mg/kg with no improvement.

### 2.1 Methods (Differential Diagnosis, Investigations and Treatment)

Upon examination the patient was afebrile; his left knee (infrapatellar) was severely tender, hot and there was limitation in active and passive range of motion with no redness or any skin changes. The patient was able to walk but had a mild left‐sided limp, in which he could not bear his weight on the left leg. Hip examination was unremarkable, showing full active and passive range of motion with a negative FABER test and no signs of inflammation. The remainder of the physical examination was normal. Depending on the full musculoskeletal exam, there was a high suspicion of tibial pathology. The patient's leukocyte count was normal, with mildly elevated inflammatory markers, including erythrocyte sedimentation rate (ESR) and C‐reactive protein (CRP). Other laboratory parameters, including kidney and liver function tests, and electrolytes, were unremarkable (Table 1).

**TABLE 1:** Summary of the patient's laboratory investigations on admission.

Test	Result (at admission)	Interpretation	Result (on follow‐up)
Leukocyte count	8.3 × 10^9^/L	Normal	8.3 × 10^9^/L
ESR	37 mm/h	Slightly elevated	5 mm/h
C‐reactive protein	23 mg/L	Slightly elevated	9 mg/L
Creatinine	0.6	Normal	N/A
BUN	12	Normal	N/A
ALT	35	Normal	N/A
AST	30	Normal	N/A
Sodium	139	Normal	N/A
Chloride	105	Normal	N/A
Potassium	4	Normal	N/A

**FIGURE 1:**
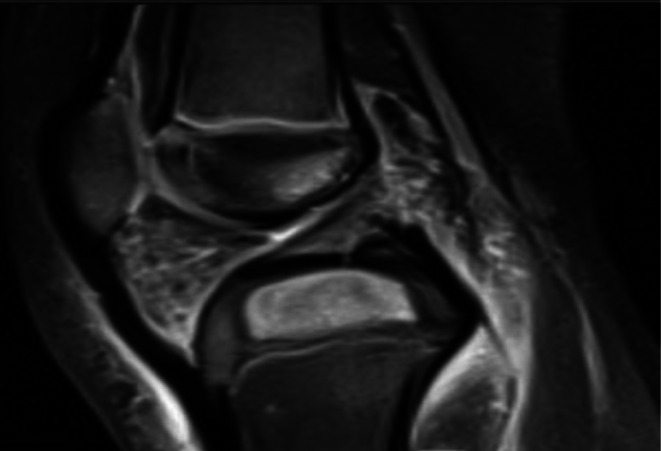
MRI showing an oval, heterogeneous enhancing lesion in the proximal tibial epiphysis.

The patient was admitted to the hospital for further management and comprehensive evaluation by both orthopedic and rheumatology specialists. Upon admission, a bilateral hip and knee x‐ray was done which showed soft tissue swelling on the left knee; otherwise, it was unremarkable. A left knee ultrasound was done and showed that there were mild subcutaneous focal inflammatory changes seen in the anteroinferior aspect of the infra‐patellar region without significant joint effusion. A hip ultrasound was done and showed widening in the joint space with no effusion or collection which has no significance. Cefazolin was started while waiting for the MRI results, which were requested. MRI revealed a 2.5 cm × 1.8 cm × 1 cm oval, mass‐like lesion with heterogeneous signal intensity and enhancement located within the epiphysis of the proximal tibia (tibial plateau) (Figure 1).

**FIGURE 2:**
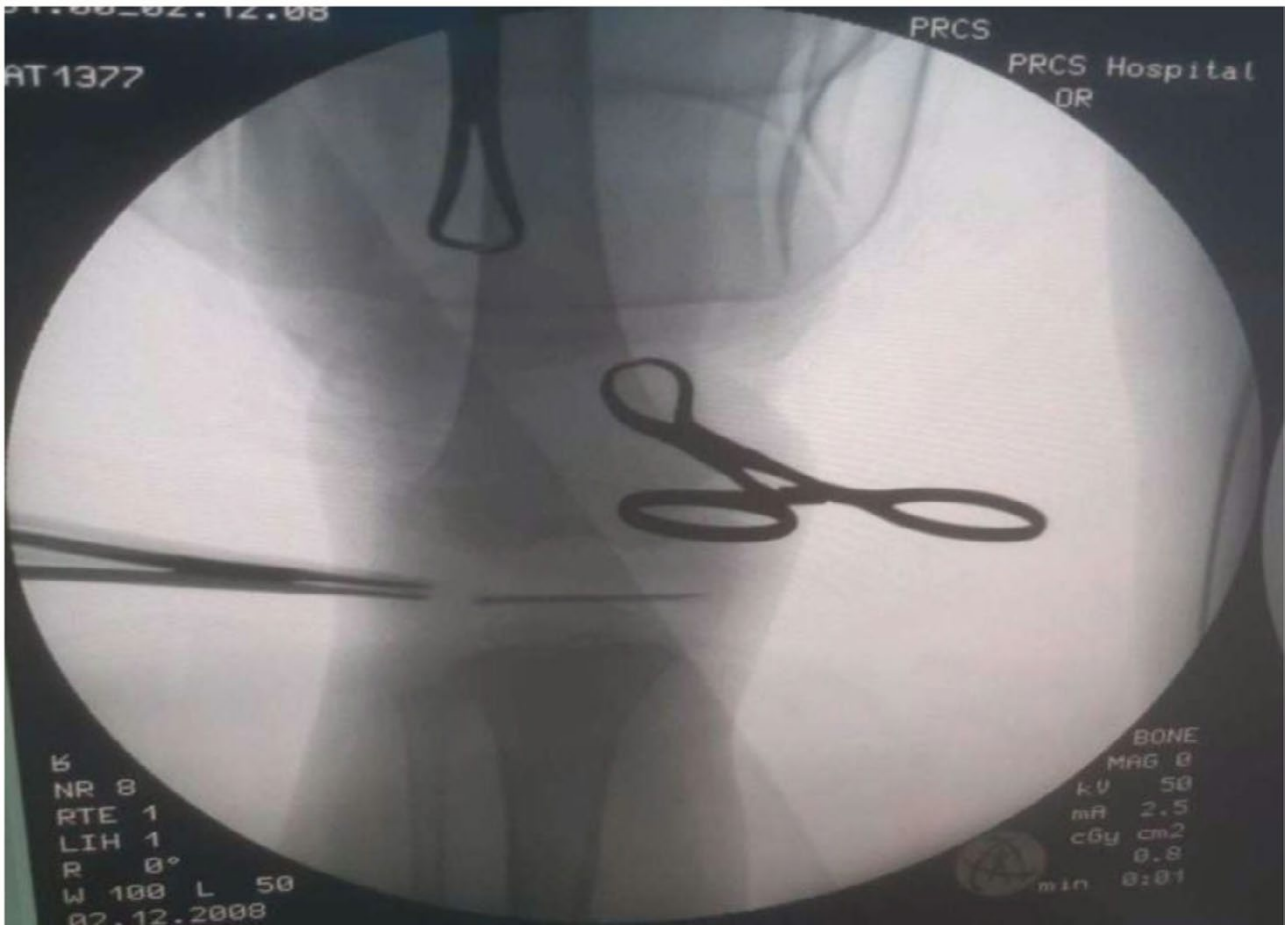
Intraoperative image of surgical drainage.

The diagnosis of subacute osteomyelitis with Brodie's abscess was favored. However, malignancy could not be excluded based on the radiologic findings, and surgical drainage was therefore undertaken (Figure 2) with biopsy and culture.

**FIGURE 3:**
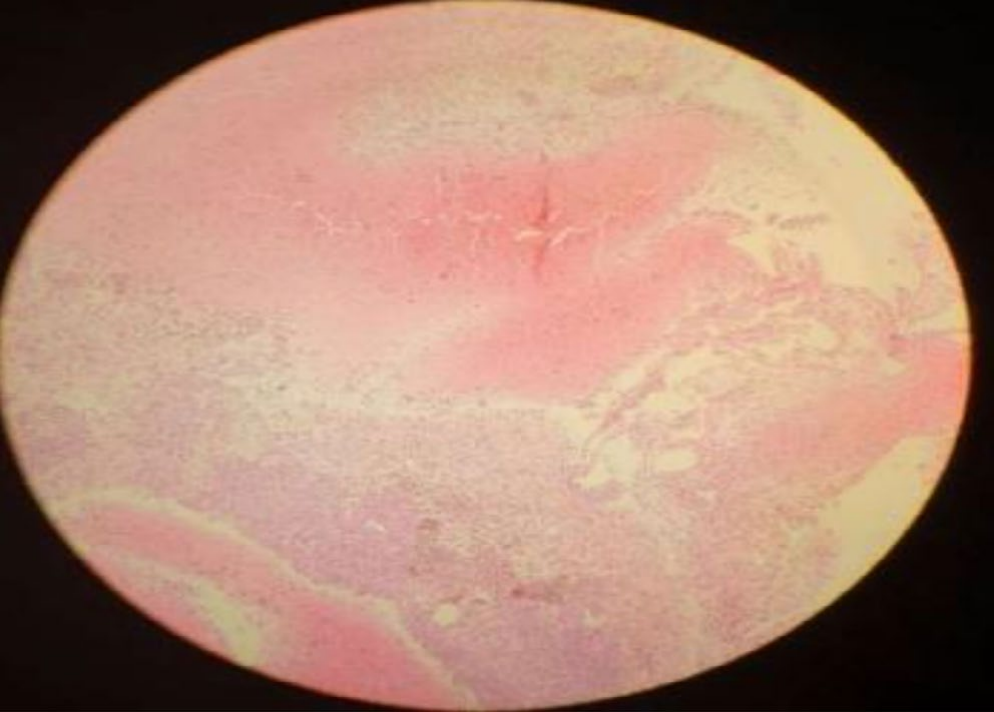
Histopathology confirming Brodie's abscess.

Culture returns negative and the biopsy result was consistent with Brodie's abscess (Figure 3). Initially, the patient continued Cefazolin for 7 days; vancomycin 500 mg IV every 12 h was added empirically to provide coverage for possible methicillin‐resistant *Staphylococcus aureus* . Upon discharge the patient was maintained on vancomycin with a good range of motion, afebrile, clean wound, CRP negative and the last ESR was 30.

The patient is advised to do regular follow‐up for at least 6 months; the last visit to the pediatric clinic showed marked improvement with normal x‐ray and lab values (Table 1).

## 3 Conclusion and Results

We reported a case of Brodie's abscess in the metaphysis of the left proximal tibia with initial presentation of bilateral hip pain. Although it is very rare, Brodie's abscess can present with referred pain as initial presentation which was shown in our case. Depending on hip physical examination and laboratory findings the plan was to send the patient home. But due to a wide differential diagnosis, which includes malignancy, further evaluation was done, and it turned out that our patient needed surgical drainage due to Brodie's abscess which was diagnosed by MRI and confirmed by biopsy.

## 4 Discussion

### 4.1 Overview of Brodie's Abscess

Brodie's Abscess is a rare condition presenting as a subacute type of bone infection leading to localized pus collection [1]. It occurs when the host defenses and the germs are equally matched, resulting in a walled‐off abscess which is responsible for reducing the systemic reaction. Both direct local bacterial invasion and hematogenous transfer of organisms to bone can result in an intraosseous infection [1, 6]. The bacteria enter the bone through a damaged area elsewhere in the body, such as a skin pustule, furuncles, infected blisters, or they enter the bone as a result of an infection in another organ system. Also, it can be caused by routine activities such as brushing teeth [11]. Frequently, the infected focus remained undetected.

### 4.2 Epidemiology

According to a systemic review of reported cases comprising a total of 70 articles (including 407 patients), Brodie's Abscess is typically seen in a young population with a median age of 17 years with minimal incidence in adult populations [6]. Sex ratios vary but in general males are affected slightly more often than females (M: F, 2.1:1) [6].

### 4.3 Clinical Presentation

Localized Pain is the most common complaint in most patients 98% accompanied by swelling in 53% of patients. 84% of patients were afebrile [6, 12]. However, our patient complained of fever and vomiting, and the hip pain was referred pain.

### 4.4 Diagnostic Challenges and Imaging

**FIGURE 4:**
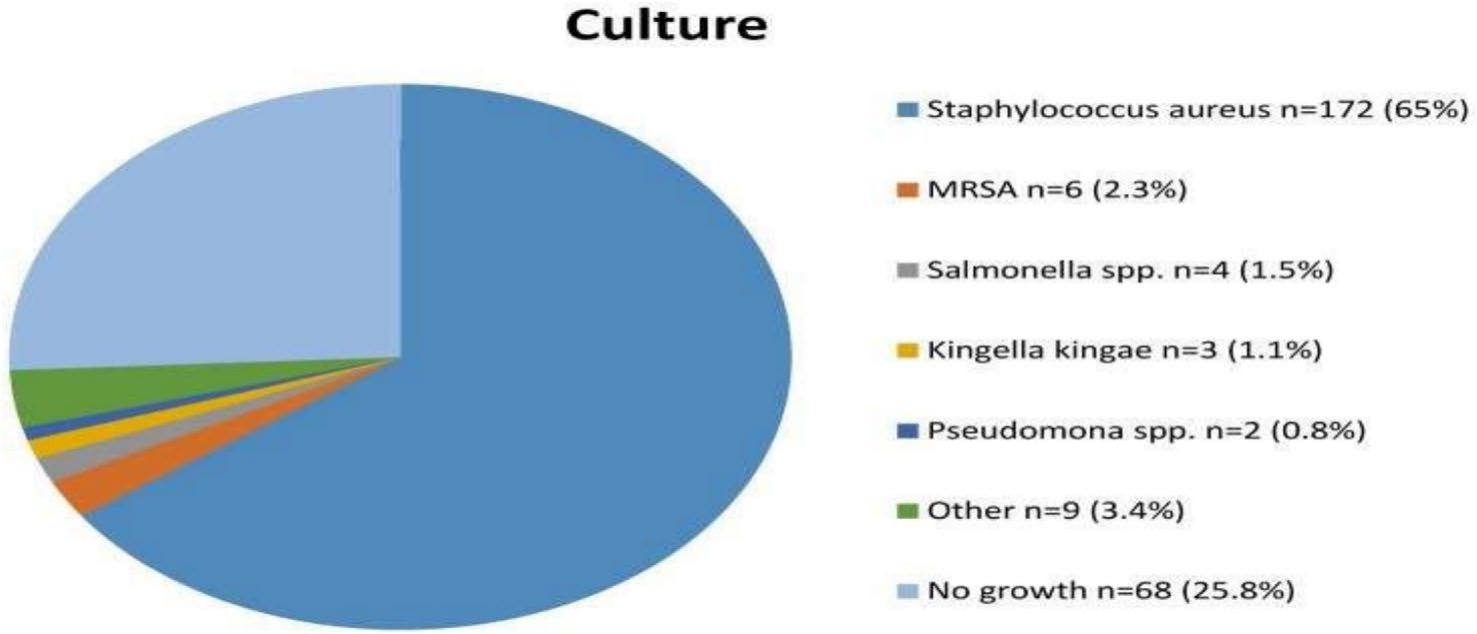
Distribution of culture results.

Symptoms of subacute osteomyelitis are nonspecific; an accurate diagnosis is sually delayed (a median of 12 weeks before diagnosis was established). The tibia (48.6%) and femur (31.1%) were mostly involved. The result of all cultures is presented in (Figure 4) [6, 13]. Given the nonspecific feature of Brodie's abscess, a high suspicion index is required along with confirmatory biopsy and MRI which are the diagnostic modalities of choice for Brodie's abscess, while the inflammatory markers ESR, CRP, and CBC have a limited role in the diagnosis [13]. ESR (55.4%) and CRP (77.5%) were mostly normal or slightly elevated [6], as in our case. Our patient was diagnosed with Brodie's abscess according to the imaging modalities (x‐ray, MRI) and biopsy to rule out other differential diagnoses like malignancy.

### 4.5 Management and Prognosis

The management of Brodie's abscess is still a controversial [6]; however, the treatment mainly involves a combination of surgical and antibiotic therapy [1]. The choice of surgery depends on tumor size and location [14]. Empirical coverage for *Staphylococcus aureus* is usually advised, and agents like cefazolin, oxacillin, or vancomycin are used when MRSA is diagnosed or suspected [15]. In the case of small, well‐localized abscesses with confirmed diagnosis and close follow‐up, the conservative management with antibiotics only option might be considered [1], but in such cases, higher recurrence rates are experienced. As a whole, the combined surgical and medical management leads to excellent prognosis and full functional recovery in most patients [6].

## Author Contributions

**Suleiman Sbeih:** writing – original draft, writing – review and editing. **Yazan Ahmad:** writing – original draft, writing – review and editing. **Mohammed Boji:** writing – original draft, writing – review and editing. **Asma Mahajnah:** writing – original draft, writing – review and editing. **Fawzi Abunejma:** writing – original draft, writing – review and editing. **Mohammad Alashqar:** software, validation, writing – original draft, writing – review and editing.

## Ethics Statement

Informed consent was obtained from the patient's parents. Our institution does not require ethical approval for case reports.

## Consent

Written informed consent was obtained from the patient's parents for publication of this case report and the accompanying images.

## Conflicts of Interest

The authors declare no conflicts of interest.

## Data Availability

The data supporting the findings of this case report is not available due to privacy and confidentiality restrictions.
